# In Situ Monitoring of Powder Bed Fusion Homogeneity in Electron Beam Melting

**DOI:** 10.3390/ma14227015

**Published:** 2021-11-19

**Authors:** Marco Grasso

**Affiliations:** Department of Mechanical Engineering, Politecnico di Milano, 20156 Milan, Italy; marcoluigi.grasso@polimi.it

**Keywords:** electron beam melting, in situ sensing, in situ monitoring, powder bed, machine vision

## Abstract

Increasing attention has been devoted in recent years to in situ sensing and monitoring of the electron beam melting process, ranging from seminal methods based on infrared imaging to novel methods based on backscattered electron detection. However, the range of available in situ monitoring capabilities and solutions is still quite limited compared to the wide number of studies and industrial toolkits in laser-based additive manufacturing processes. Some methods that are already industrially available in laser powder bed fusion systems, such as in situ detection of recoating errors, have not yet been investigated and tested in electron beam melting. Motivated by the attempt to fill this gap, we present a novel in situ monitoring methodology that can be easily implemented in industrial electron beam melting machines. The method is aimed at identifying local inhomogeneity and irregularities in the powder bed by means of layerwise image acquisition and processing, with no external illumination source apart from the light emitted by the hot material underneath the currently recoated layer. The results show that the proposed approach is suitable to detect powder bed anomalies, while also highlighting the link between the severity of in situ detected errors and the severity of resulting defects in the additively manufactured part.

## 1. Introduction

The quality and performance of high-value-added parts produced via powder bed fusion (PBF) processes, either laser- or electron beam-based, are known to be affected by a large variety of factors. Several sources of poor quality can be predicted and tackled by properly selecting, controlling, and optimizing powder properties, process parameters, build design choices, and machine calibration settings. However, a variety of other events and process dynamics that are difficult or impossible to model and predict may lead to stochastic variations and detrimental effects on the final quality and conformity of the part. Because of this, a whole body of scientific literature and industrial research has been devoted to in situ measurement and monitoring methods suitable to detect the onset of process defects and the origination of unstable process conditions, exploiting sensor data acquired during the process. Recent studies reviewed the literature devoted to in situ monitoring of PBF and summarized the most recent advances and achievements in this field [1,2].

One category of methods involves the in situ measurement and characterization of layer properties, exploiting so-called “powder bed cameras”, i.e., off-axially mounted cameras that acquire one or more pictures on a layer-by-layer basis. Images captured just after the powder recoating operation allow the detection of possible anomalies in the powder bed, such as incomplete spreading, recoater hopping or streaking patterns, contaminations caused by debris or deposited spatters, and superelevated edges [3,4,5]. Every departure from a regular and homogeneous powder bed is known to be a critical source of defects in the part. Powder recoating errors may also be responsible for defect propagation from one part to another within the same build and from one layer to another. Because of this, almost all laser PBF (L-PBF) system developers currently provide their machines with embedded powder bed cameras and, in some cases, with basic automated powder bed anomaly detection capability, albeit commonly limited to macroscopic errors [1,2]. Images acquired after the melting phase, once the solidification of the scanned area has occurred, may be used for different aims, such as detecting undesired surface irregularities in the solidified layers, as possible sources of internal and surface defects [6,7,8,9,10], or signaling possible deviations with respect to the nominal shape in the layer, as evidence of geometrical errors [11,12,13,14,15]. Alternative sensing methods have also been presented, including fringe projection combined with single or multiple cameras for surface topography reconstruction [16,17,18,19] and high-spatial-resolution scanning sensors installed onto the recoating arm [20,21]. These alternative methods showed the potential to overcome some of the limitations of more traditional powder bed imaging approaches, but they have not yet achieved industrial adoption in production environments.

**Figure 1:**
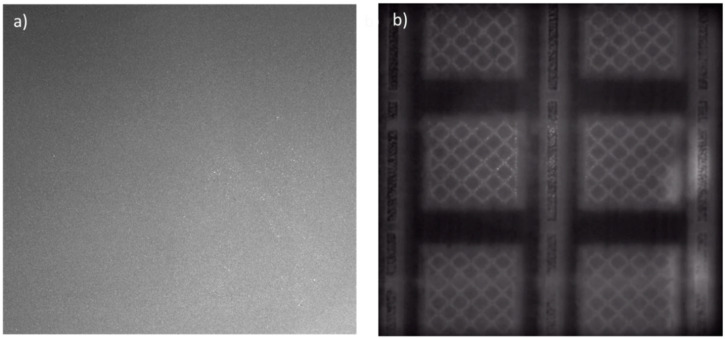
Example of defect-free post-recoating powder bed images in L-PBF (**a**) and EB-PBF (**b**).

The capability to detect local inhomogeneity in the powder bed has been explored mainly (or almost exclusively) in L-PBF, whereas almost no study has been devoted to electron beam PBF (EB-PBF, also known as electron beam melting or electron beam selective melting). One main reason is the different nature of powder bed images acquired in L-PBF compared to those acquired in EB-PBF. An example is shown in Figure 1. The two panels of Figure 1 show powder bed images acquired with an off-axially mounted camera in the visible range just after powder recoating in one layer in L-PBF (Figure 1a) and EB-PBF (Figure 1b).

Figure 1a is a typical example of a post-recoating image in L-PBF, where no powder recoating error is present. Apart from smooth pixel intensity variations caused by nonuniform lighting within the build area, the homogeneous pattern shown in Figure 1a aids the detection of discontinuities and local deviations. The nature of the powder bed image shown in Figure 1b is quite different. In EB-PBF, a layerwise preheating operation is carried out before starting the selective melting of the material. This operation is performed by means of high-speed electron beam scanning of the top surface, resulting in a quite homogeneous preheating temperature for the whole duration of the build and at all build heights. Such an operation raises the powder bed temperature up to a level that is sufficient to pre-sinter the powder and counteract disruptive phenomena related to excessive surface charge accumulation (also known as “smoke” events). The preheating temperature may range from hundreds of degrees Celsius to more than 1000 °C, depending on the manufactured material. If a color image was acquired, it would show a powder bed of intense red color, because of such high temperature. When a new layer of cold powder is spread over the previous one, the underneath temperature is so high that the hotter regions of the previous layer (i.e., the bright areas in Figure 1b) are still well visible through the new one. Hot areas consist of solidified regions and their surroundings, i.e., areas of the build that underwent successive melting, preheating, and post-heating phases (post-heating may be applied in some layers after the melting phase to balance the heat input provided to the material, with the aim of keeping such input as constant as possible during the entire build).

The nature of power bed images like the one in Figure 1b makes the design of a robust and automated method to detect anomalies in the powder spread more challenging than in L-PBF, while also considering that the extension, shape, and intensity of underneath hot areas may change from one layer to another. A preliminary approach to detect powder bed irregularities in EB-PBF was proposed in [22]. A different approach relying on the use of fringe projection was proposed in [18,23]. The proposed system involved a single-view architecture, i.e., a monocular vision. The fringe projection measurements were taken both after powder recoating and after the melting phase. The most recent study from the same authors [19] showed that, by combining the fringe projection approach with an intelligent technique to deal with large variations of the reflective characteristics of the surface, enhanced topographic results could be achieved with a quite reduced measurement time. The method was tested by reconstructing both the powder bed and the solidified layer.

Another approach involves the use of so-called “electronic imaging”, i.e., an imaging approach that relies on backscattered and secondary electrons generated when the electron beam scans the build area, similarly to the principle adopted in scanning electron microscopy. Wong et al. [24] demonstrated the feasibility of the technique in generating raw images where each pixel value was proportional to the signal strength of backscattered and secondary electrons. More recently, the same authors showed that the method could be used for geometrical reconstruction of solidified layers [25] and for material contamination detection [26]. Arnold et al. [27] extended and tuned the method, showing the capability to achieve a quite high spatial resolution in the measurement of the solidified layer. Arnold et al. [28] demonstrated that the same approach could be generated during the melting phase, leading to an “in operando” monitoring capability. Although being potentially suitable to characterize the homogeneity of the powder bed, the electronic imaging method has so far been studied mainly for the characterization of the solidified material in the layer. Moreover, acquiring an electronic image of the powder bed just after the powder recoating operation would require extra time, during which the electron beam is used to scan the build area for image generation.

Our study presents an automated powder bed monitoring methodology applicable to EB-PBF, which relies on a camera and no external illumination source (apart from the light emitted by the hot surface of the build). One main advantage is the ease of adoption on industrial EB-PBF machines, possibly exploiting the already installed hardware equipment. Another advantage compared to fringe projection regards the lower dimensionality of layerwise images compared to actual topographic measurements, which makes data collection and storage more efficient. Furthermore, the method requires no extra time for powder bed measurement, unlike other techniques mentioned above.

The proposed method is aimed at bridging the gap between consolidated powder bed monitoring methods in L-PBF and the current lack of such methods in EB-PBF. It combines different image processing steps to isolate possible contaminations of the powder bed, as well as powder spread errors caused by a damaged recoater. The present study extends the preliminary idea presented in [22] and demonstrates its effectiveness through experiments carried out on an industrial EB-PBF system. It also presents a characterization of the effect of recoating errors on the final quality of additively manufactured parts, showing to what extent the severity of anomalies detected in situ may affect the consequent severity of volumetric and geometrical defects in the part.

Section 2 presents the proposed methodology and the associated sensing setup. Section 3 presents the case study and the main results. Section 4 provides a brief discussion of the method’s performance and its industrial applicability. Section 5 concludes the paper.

## 2. Materials and Methods

**Figure 2:**
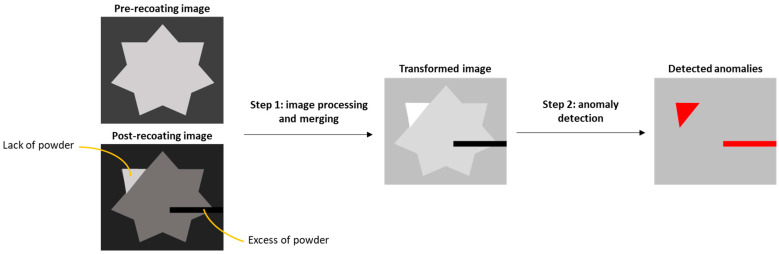
Scheme of the proposed approach.

A scheme of the major steps involved in the proposed approach is shown in Figure 2. The input consists of a couple of images per layer, namely, the pre- and post-recoating powder bed images. The first step includes preprocessing operations that lead to a merging of the information content enclosed in the two input images. Then, the transformed image is further processed in the second step, where a transfer function applied to the pixel intensity histogram leads to the automatic segmentation of areas corresponding to anomalous powder recoating. Thus, the proposed approach allows signaling an alarm combined with a 2D representation of the anomaly location.

The proposed sensing setup for pre- and post-recoating image acquisition is presented in Section 2.1. The main steps of the methodology are presented and discussed in Section 2.2 and Section 2.3.

### 2.1. Powder Bed Imaging Setup

**Figure 3:**
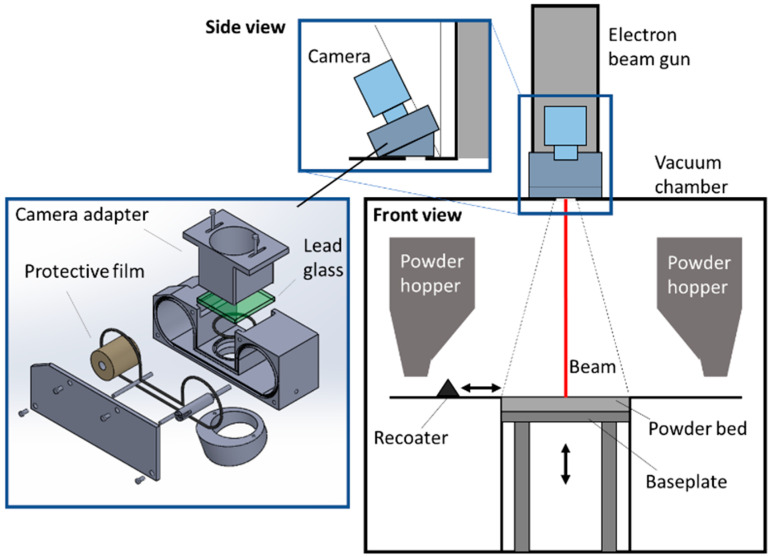
Customized camera mounting with rolling protective film on an Arcam A2 system.

The proposed sensing setup takes advantage of the viewport on the top of the build chamber, which is available in industrial EB-PBF systems. An example is shown in Figure 3, where the machine configuration refers to the Arcam A2/A2X system.

Any machine vision equipment for EB-PBF process monitoring should be protected from metallization. In this study, we present a custom camera mounting device where a rolling film is used to avoid camera lens metallization. As an alternative, a mechanical shutter can be used, which is activated only during image acquisition. The custom camera mounting proposed here includes a 45 mm width Kapton^®^ Type 100 HN rolling film and a 60 × 60 × 5.5 mm lead glass to shield the X-ray emissions. The protective film is shifted once per layer before image acquisition. A high-spatial-resolution camera equipped with a sensor in the visible range was used (see Section 3 for specifications). A near-infrared filter may also be used as a possible extension of the proposed approach to reduce the spectrum bandwidth and the dynamic range.

The image acquisition should be synchronized with the process phases and with the recoater displacement. The pre-recoating image is acquired at the end of the melting, contouring, and post-heating phases of the previous layer, i.e., when the production of the previous layer is over. In addition, a hardware trigger is generated to take a powder bed picture at the end of each recoater stroke. In EB-PBF, multiple (commonly three) recoating passes are usually applied to obtain a homogeneous powder bed. A picture can be taken at the end of each recoater pass. In this study, we advocate the use of the image acquired after the last recoating pass only, as it is the one capturing the actual layer condition before the successive processing phase. This image is saved and used as the post-recoating image, i.e., the second input for our proposed approach.

The experimentation presented in this study was carried out by producing Ti_6_Al_4_V specimens with an Arcam A2 system (GE Additive, West Chester, PA, USA), using default process parameters provided by the machine vendor. A Ti_6_Al_4_V extra low interstitial (ELI) powder with particle size in the range 45–106 µm was used.

### 2.2. Image Preprocessing and Merging

As shown in the example in Figure 1, a robust identification of areas where an anomalous powder recoating occurred may be challenging if the post-recoating image alone is analyzed. Combining the pre- and post-recoating images, instead, enhances the capability to distinguish natural patterns from anomalous ones.

Generally speaking, three kinds of anomalies may occur, leading to different patterns in the image. The first consists of a local lack of powder. Depositing no powder or an insufficient amount of powder on a surface that appears bright because of its high temperature results in a bright area in the post-recoating image. In this area, pixel intensities are expected to be equal or very close to pixel intensities observed in the same area in the pre-recoating image, because of the small amount of powder deposited on top of it. The second type of anomaly consists of an excess of powder. In this case, an area where the powder layer is thicker results in a darker region compared to other areas of the same image, because of a thick layer of cold powder. If excessive powder is deposited over a previously melted region, pixel intensities will experience a higher reduction compared to pixels belonging to properly recoated areas. The third type of anomaly consists of a contamination of the powder bed, i.e., the presence of debris. This kind of anomaly is expected to yield an effect in post-recoating images that is analogous to the one of a local excess of powder, as the debris will cover and mask the underneath material. Therefore, by combining and comparing the spatial pixel intensity information enclosed in pre- and post-recoating images, all types of anomalies may be detected.

The first step consists of three sequential image processing operations:

- Correction of the image perspective error. This operation can be applied during the camera calibration stage. The reader is referred to [29] for an overview of calibration methods commonly applied in machine vision.
- Image filtering, applied to both input images in each layer. The aim is to reduce the noise of the image, smoothing pixel-wise intensity variations. A high image noise is caused by the lack of an external illumination source, which imposes the adoption of a sufficient integration time combined with a sufficient sensor sensitivity enhancement. Is this study, we advocate the use of a median filter applied to both pre- and post-recoating images. It is a nonlinear filter that is known to be quite effective for a simultaneous reduction in image noise and preservation of contours [30]. The pixel intensity in location (x,y) is replaced by the median of pixel intensities in a neighborhood of m×m pixels. A larger value of m denotes more intense smoothing, with a loss of image details. A smaller value of m denotes a higher preservation of edges but lower noise filtering. In this study, we adopted a median filtering with m=5, but the parameter m can be easily tuned during the image calibration phase to be carried out just once when exploiting sample images.
- Image transformation. Let $I_{pre}$ be the filtered pre-recoating image in one layer and $I_{post}$ be the filtered post-recoating image in the same layer. Let $i_{pre}\left(x,y\right)$ and $i_{post}\left(x,y\right)$ be the intensity of a pixel in location (x,y) of $I_{pre}$ and $I_{post}$, respectively. In a grayscale 8 bit image, the pixel intensity is an integer value in the range 0–255, where 0 corresponds to the lowest intensity (black) and 255 corresponds to the highest intensity (white). The proposed way to merge the information in the two input images consists of applying the following transformation:
  (1) $$I_{tr}=\frac{\left|I_{post}-I_{pre}\right|}{I_{pre}},$$
  where $I_{tr}$ is a transformed image whose pixel intensities, $i_{tr}\left(x,y\right)$, belong to the range 0–1.

The rationale for this transformation is the following: $I_{post}$ is such that $i_{post}\left(x,y\right)\leq i_{pre}\left(x,y\right)$, because of the powder recoating operation (the reader is reminded that the cold powder in the newly recoated powder bed looks dark because of its low temperature) and the slow cooling of the underneath layer. By computing the absolute difference between the two images and normalizing the result dividing by the pixel intensities in $I_{pre}$, two extreme conditions can be met. The first consists of pixels such that $i_{tr}\left(x,y\right)=0$. This can occur in regions where there was no difference between the two images in location (x,y), i.e., in regions where no powder recoating occurred. The second consists of pixels such that $i_{tr}\left(x,y\right)=1$. This can happen in regions where $i_{post}\left(x,y\right)=0$, i.e., where the thickness of the powder is so high that it results in a perfectly black pixel. Thus, a defective powder recoating is expected to generate pixel values that are close (or equal) to 0 and 1, meaning a lack or an excess of powder, respectively. Intermediate pixel intensities, instead, are representative of normal (error-free) powder recoating, where intensity variations are mainly due to local temperature differences in the underneath layer and to natural fluctuations in the powder bed thickness.

It is worth noticing locations where $i_{pre}\left(x,y\right)=0$ results in $i_{tr}\left(x,y\right)=inf$. These pixels are deemed of no interest, as they correspond to a dark (cold) background where no previous melting or pre/post-heating was performed; hence, irregularities in the powder recoating in that location would fall outside the printed region. These pixels, if any, can be excluded from subsequent analysis by replacing them with a “not a number” (NaN) label.

The transformed image, $I_{tr}$, is the input to the successive step of the proposed algorithm.

### 2.3. Automated Anomaly Detection

Relying on the fact that extreme values of pixel intensity $i_{tr}\left(x,y\right)$ correspond to potential anomalies in the powder bed, the second step of the proposed approach consists of applying a transfer function to the histogram of pixel intensities in $I_{tr}$ to enhance the isolation of those anomalies. Such a transfer function is thought to associate a high value with the right and left tails of the pixel intensity distribution and a low to null value with the central portion of the distribution. By applying such a function to $i_{tr}\left(x,y\right)$, a new image called $I_{tr*}$ is generated, where both a lack of powder and an excess of powder anomalies result in high-intensity regions, whereas all other areas appear as a low-intensity background.

Different ways to define such a transfer function can be envisaged. The methodology proposed in this study is described below. A sensitivity analysis with respect to the choice and definition of this function is discussed in Section 3.

**Figure 4:**
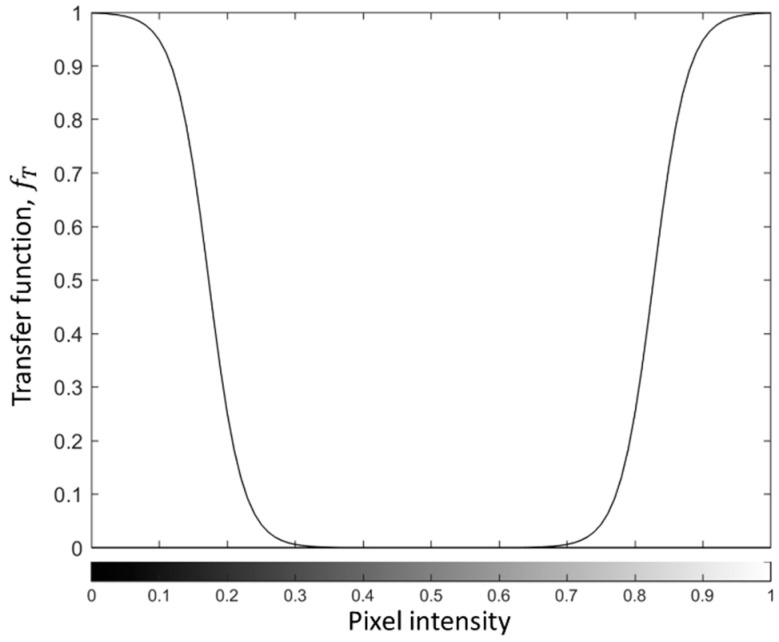
Transfer function for the conversion of the image $I_{tr}$.

The image $I_{tr*}$ is an image of the same size as $I_{tr}$, whose pixel intensities $i_{tr*}\left(x,y\right)$ are given by

(2) $$i_{tr*}\left(x,y\right)=f_{T}\left(i_{tr}\left(x,y\right)\right),$$

where the function $f_{T}\left(\cdot \right)$ is a reverse sigmoid function for $i_{tr}<0.5$ and a sigmoid function for $i_{tr}\geq 0.5$, as shown in Figure 4. The function $f_{T}\left(\cdot \right)$ transforms pixel intensities $0\leq i_{tr}\leq 1$ into $0\leq i_{tr*}\leq 1$. It can be defined as follows:

(3) $$\begin{array}{l} f_{T}\left(i_{tr}\right)=\frac{1}{1+ae^{bi_{tr}}},\;i_{tr}<0.5,\\ f_{T}\left(i_{tr}\right)=\frac{1}{1+ae^{b\left(1-i_{tr}\right)}},\;i_{tr}\geq 0.5, \end{array}$$

where a and b are two weight parameters that determine the shape of the function; a is just a centering parameter to transform intensities in the range 0–1 to intensities in the same range, whereas b is a parameter that controls the slope of the sigmoid function. The function shown in Figure 4 is such that $a=1e^{-3}$ and b=40, resulting in inflection points at $i_{tr}<0.2$ and $i_{tr}>0.8$, and $i_{tr*}$ values that quickly drop to zero in the range $0.3<i_{tr*}<0.7$. The reader is referred to Section 3 for a discussion on the influence of the expression and parameters of the function $f_{T}\left(\cdot \right)$ on the performance of the proposed approach.

By transforming the image $I_{tr}$ by means of the function $f_{T}\left(\cdot \right)$, it is possible to get rid, to a large extent, of geometry-dependent patterns. Indeed, in the absence of powder bed anomalies, $I_{tr*}$ is expected to be a quite uniform image whose pixel intensities are very close (or equal) to zero in every location. This makes the resulting image, $I_{tr*}$, suitable to detect anomalies as local deviations from a uniform and low-intensity image.

The last step consists of defining an alarm rule to automatically signal groups of pixels belonging to the $I_{tr*}$ image, which correspond to powder bed anomalies. To this aim, a small set of powder bed images where no recoating error was observed can be used as a training set. Thanks to the image transformations mentioned above, these training images can be gathered from previous builds regardless of the geometry of printed parts; hence, the output of the training phase can be applied to future builds even if they are different from each other. Let M be the number of training images and let $i_{tr*}$ be a vector that includes all pixel intensities, $i_{tr*}$, for all pixels of all M images $I_{tr*}$ included in the training set. If N is the number of pixels in each $I_{tr*}$ image, the length of the $i_{tr*}$ vector is MN. Then, a pixel $i_{tr*}\left(x,y\right)$ belonging to any newly acquired $I_{tr*}$ image is labeled as an anomalous pixel if

(4) $$i_{tr*}\left(x,y\right)>k_{\alpha },$$

where $k_{\alpha }$ is the 100(1−α)% percentile of the empirical distribution of the training vector $i_{tr*}$, being $\alpha =\alpha^{\prime }/N$, where $\alpha^{\prime }$ is the familywise target false alarm rate. As commonly done in statistical process monitoring applications, the target false alarm rate can be set at $\alpha^{\prime }=0.0027$ [31].

## 3. Results

### 3.1. Case Study with Damaged Recoater

This section presents a real case study where powder recoating errors were induced on purpose in order to evaluate the performance of the proposed approach.

**Figure 5:**
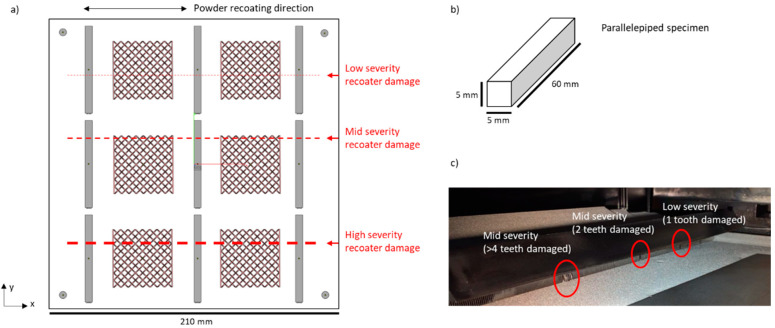
(**a**) Top view of the build area; (**b**) size of the parallelepiped specimens included in the build; (**c**) purposely inserted damage in the powder recoating system.

Two different types of parts were included within the 210 × 210 mm build area, as shown in Figure 5. The first consisted of nine parallelepiped specimens of size 5 × 5 × 60 mm. The second consisted of six squared lattice structures, with thin vertical walls on the right and left side. Four dummy cylinders were built vertically in the four corners of the build area, as commonly done in EB-PBF, to induce a homogeneous preheating temperature across the area. The combination of different geometries within the same build was used to generate a complex pixel intensity pattern in powder bed images, but a deeper analysis of the defectiveness induced by the powder recoating errors was devoted mainly to the parallelepiped specimens.

The powder recoating errors were induced as shown in Figure 5c, by artificially damaging the thin metal teeth of the recoater with different severities and in different locations. The recoater consists of two parallel rows of metal teeth. It spreads the powder from left to right and from right to left, with three consecutive passes per layer. The following damages were inserted:

- Low-severity damage: only one tooth along one row was manually warped;
- Mid-severity damage: two teeth per row were removed;
- High-severity damage: 2 two per row were removed, and the neighboring teeth were manually warped.

After the process, two parallelepiped specimens affected by mid- and high-severity powder bed discontinuities were inspected by means of X-ray computed tomography (CT). A North Star Imaging X25 micro X-ray CT system (North Star Imaging, Rogers, MN, USA) was used, and the measurements were performed with a voxel size of 7.62 µm.

In situ powder bed images were acquired by means of the setup described in Section 2. The main camera specifications are shown in Table 1. The field of view of the camera covered up to 90% of the build area, but this can be increased by modifying the aperture on the top of the heat shield. In addition to the acquisition of pre- and post-recoating images, videos were acquired with the same camera both during each powder recoating operation and during the subsequent preheating, melting, and post-heating phases. The aim was to gather a sufficient amount of information to better interpret and characterize the occurred anomalies and other possible stochastic phenomena.

In order to determine the control limit, $k_{\alpha }$, 20 images from another build previously performed with the same EB-PBF system, the same process parameters, and the same Ti_6_Al_4_V powder were used. The build composition was different, consisting of lattice structures only. The aim was to highlight that the proposed method is quite insensitive with respect to the shape of parts produced in the build, enabling the use of builds different from the one that is currently monitored for training purposes.

**Figure 6:**
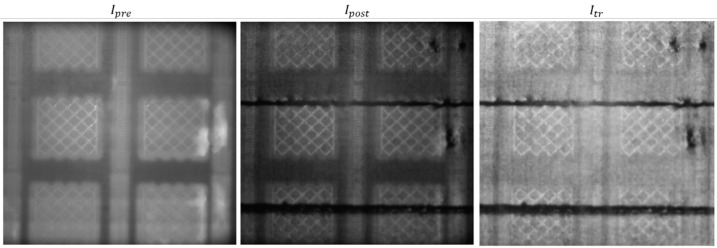
Example of pre- and post-recoating image in one layer where the mid- and high-severity damages of the powder recoating images produced visible discontinuities; the right panel shows the transformed image $I_{tr}$.

Figure 6 shows an example of pre- and post-recoating images, $I_{pre}$ and $I_{post}$, as well as the corresponding transformed image, $I_{tr}$. Figure 6 clearly shows two dark horizontal tracks, corresponding to an excess of powder along the line where the powder recoater was damaged with mid and high severity. Evidence of the low-severity damage is instead not visible in the image. Table 1 shows that the spatial resolution was about 130 micron/pixel. Considering that one tooth of the powder recoater has a width of about 1 mm, the spatial resolution should be sufficient to capture a powder recoating error on the order of 1 mm. However, the visual inspection of the powder bed after the process confirmed no actual presence of any discontinuity associated with the low-severity damage. Indeed, one single tooth damaged was not sufficient to have any detrimental effect on the powder bed homogeneity.

Figure 6 also shows that, in addition to the horizonal discontinuities induced on purpose, some discontinuities were present on the righthand side of the image (they are clearly visible are darker spots in the transformed image, $I_{tr}$). The images in Figure 6 represent an example of what happened during the production of the first few layers of the parallelepiped specimens, once the supports were completed. During the deposition of the first layers, not only were the purposely created discontinuities more evident than in following layers, but other discontinuities were generated on the righthand side, whenever the last recoating pass was performed from right to left. In those locations, for the duration of few consecutive layers, a slower cooling transitory was observed after the melting phase, as can be seen from Figure 6 (left panel); the brighter regions on the righthand side of the image correspond to areas that stayed hot for a longer time after the melting of the previous layer. All this evidence suggests that some irregularities in the powder deposition occurred in the first few layers in that region, affecting the local thermal history.

**Figure 7:**
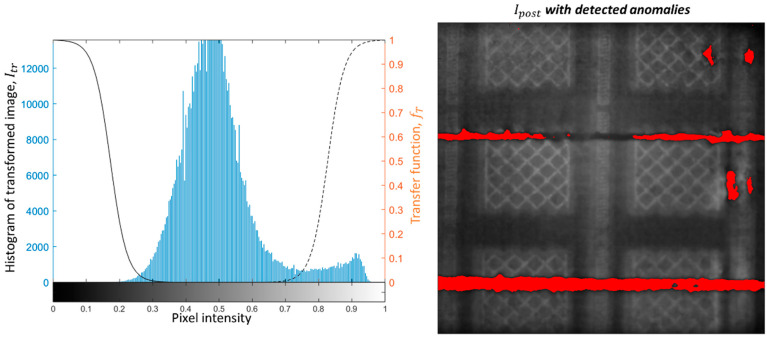
Pixel intensity histogram of the $I_{tr}$ image (**left panel**) and the result of anomaly detection (**right panel**; pixels that caused a violation of the alarm rule are shown in red and superimposed on the post-recoating image).

Figure 7 shows the pixel intensity histogram of the transformed image, $I_{tr}$, shown in Figure 6 and the corresponding anomaly detection obtained with the proposed approach (pixels that caused a violation of the alarm rule are shown in red and superimposed on the post-recoating image). Figure 7 shows that the proposed approach is suitable to detect both anomalies introduced on purpose and additional anomalies observed on the righthand side of the build area.

**Figure 8:**
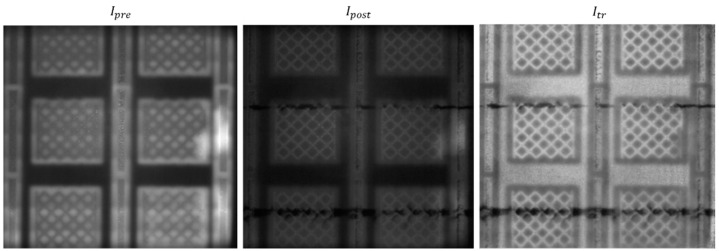
Example of pre- and post-recoating images with the corresponding transformed image $I_{tr}$.

**Figure 9:**
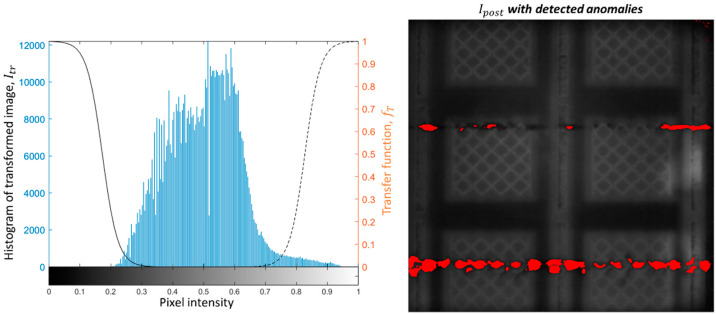
Pixel intensity histogram of the $I_{tr}$ image (**left panel**) and the result of anomaly detection (**right panel**).

Figure 8 and Figure 9 show another example from the same build, corresponding to one layer produced 1 mm above that captured in the previous figures. In this case, the purposely introduced anomalies were also detected by the method, and no false alarm was produced elsewhere. It is also worth noting the difference in the pixel intensity histograms shown in Figure 7 and Figure 9. They refer to two different layers, where pixel intensity variations may have been due to variations in the heat input to the material. Despite such a difference, the proposed approach resulted effective in detecting the mid- and high-severity anomalies. Some portions of the dark track left in correspondence of the mid-severity damage were not signaled as alarms. This was due to the pixel intensity in those portions not being sufficiently low to be distinguished from natural pixel intensities observed during the training phase.

**Figure 10:**
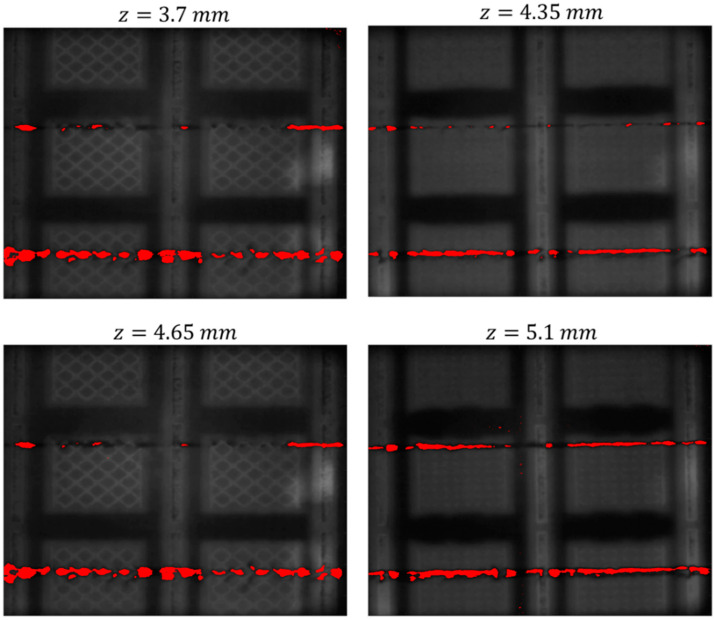
Additional examples of anomaly detection results in different layers.

Other examples of anomaly detection result in other layers of the same build are shown in Figure 10. The image in the bottom-right panel of Figure 10 also shows a few sparse pixels highlighted in red outside the purposely induced discontinuities. Since no evidence of anomalies was found by visually inspecting the layerwise image, they can be classified as false alarms.

In order to determine the performance of the proposed approach in terms of false alarms, the signaled areas in all monitored layers were visually inspected by a human expert. The following metrics were computed: (1) overall percentage of pixels where false alarms where signaled, (2) average area of connected components consisting of pixels where false alarms where signaled, and (3) maximum area of those connected components. The percentage of false alarms was computed as the overall percentage of pixels in the $I_{tr*}$ image that violated the limit in Equation (4) despite belonging to locations where no actual anomaly was present. The results associated with these three metrics are summarized in Table 2. The values of the second and third metrics are rounded to the closest integer.

Table 2 shows that the proposed approach is quite robust to false alarms, and that false alarms occurred in the form of small and sparse clusters of pixels with outlying intensity. A cluster of four pixels corresponds to an area whose equivalent diameter is 0.26 mm, whereas a cluster of 32 pixels corresponds to an area whose equivalent diameter is 0.7 mm. These areas are quite small compared to the actual size of critical anomalies that may affect the final quality of the part. As a possible extension of the proposed method, a threshold can be applied to the size of the connected component identified as an anomaly, further enhancing the robustness to false alarms.

**Figure 11:**
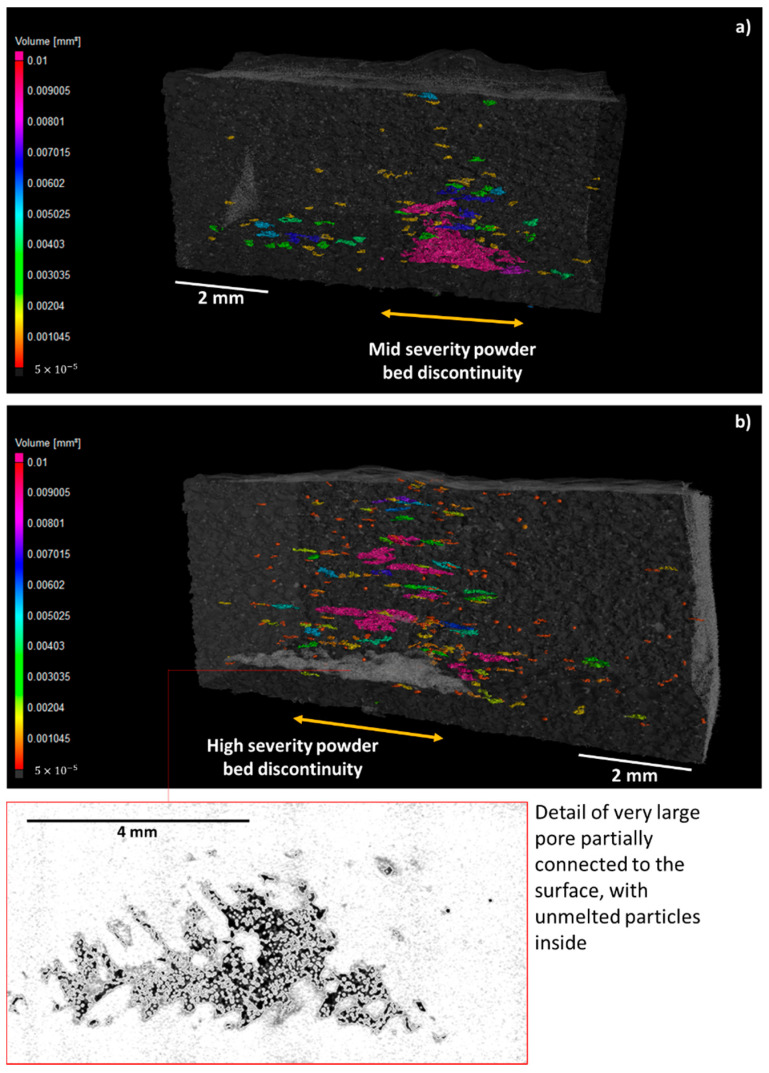
X-ray CT inspections of two specimens placed in locations of the build area where mid-severity (**a**) and high-severity (**b**) powder bed discontinuity was present.

**Figure 12:**
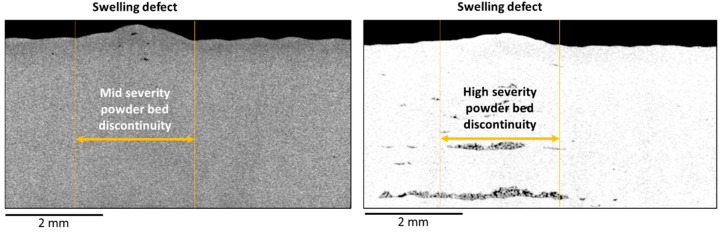
Sections of the X-ray CT reconstruction of the same specimens shown in Figure 11, which highlight the swelling defect on the top surface: specimen affected by mid severity (**left panel**) and high severity (**right panel**) powder bed discontinuity.

A further analysis was performed to investigate the effect of powder recoating discontinuities on the quality of manufactured part. Figure 11 and Figure 12 show the results of X-ray CT inspections on two specimens affects by mid-severity and high-severity powder bed discontinuity, respectively.

Due to the powder bed discontinuity, the specimens exhibited both a swelling on the top surface (Figure 12), i.e., a geometrical distortion caused by the excessive powder thickness, and a concentration of a lack of fusion pores, i.e., pores characterized by an irregular shape and the presence of unmelted powder particles within them (Figure 11). One known cause of a lack of fusion porosity is the diminished volumetric energy density provided to the material as a consequence of a thicker layer of powder [32].

Figure 11 shows a higher concentration of pores in the regions affected by the excess of powder in the layer, with largest pores (equivalent diameter > 1 mm) in the bottom portion of the specimens, where the effect of the powder recoating discontinuity is also more evident from in situ images (Figure 7). The porosity at some heights along the build direction was so severe that a partial delamination between consecutive layers was observed (as shown in detail in the right panel of Figure 11). A detailed view of a delamination that produced a large and irregular void partially connected to the outer surface is shown. The large amount of unmelted powder particles within the void is also clearly visible.

Thanks to its ability identify in situ anomalies responsible for such severe defects in the part, the proposed approach can allow the operator to stop the process, preventing a waste of material and resources in the production of scraps.

### 3.2. Other Examples

In order to evaluate the performance of the proposed approach in the presence of different build geometries and powder bed discontinuities that were not induced on purpose, the methodology was tested during the production of other parts with the same EB-PBF machine and with the same experimental settings mentioned in the previous sub-sections.

**Figure 13:**
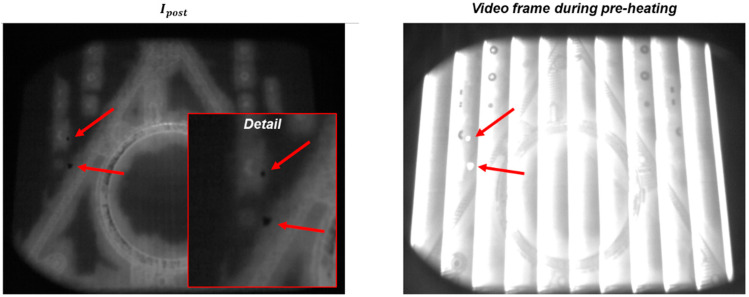
Example of powder bed contamination caused by metallization slivers: (**left panel**) post-recoating image with a detailed view of the slivers on the powder bed and (**right panel**): video frame acquired during the preheating phase, highlighting the metallization slivers that became hot spots.

One example is shown in Figure 13. In this case, a large and complex shape was produced together with a few small specimens. During the production of one layer, a detachment of portions of the metallization coating from the heat shield was observed. In EB-PBF, the metallization phenomenon results from the vaporization of metal alloying elements with a lower vaporization point. A thin layer of metallization coating is deposited on all the surfaces that surround the build area for the entire duration of the process. Sometimes, portions of this metallization may detach and fall on the powder bed, with potentially detrimental effects, as they may contaminate the powder bed and interfere with the electron beam. In the example shown in Figure 13, two small metallization slivers fell on the powder bed just after the powder recoating operation (apparently, they detached from the flaps of the heat shield). They are visible as dark spots in the post-recoating image (Figure 13, left panel). The smaller one had an equivalent diameter of about 10 pixels (1.3 mm), whereas the larger one had an equivalent diameter of about 20 pixels (2.6 mm). During preheating, they became hot spots, which stayed hot for a longer time compared to the surrounding pre-sintered powder (Figure 13, right panel). In this example, the metallization material fell on a region of the build area where no electron beam melting was performed, with no consequences for the part quality. If they had fallen over a melting zone, they could have prevented the correct melting of the material, possibly leading to a volumetric defect.

**Figure 14:**
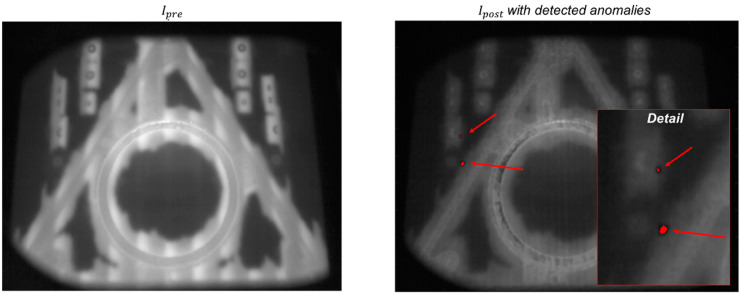
Pre-recoating (**left panel**) and post-recoating (**right panel**) images in the layer affected by the metallization contamination; the result of the anomaly detection is superimposed on the post-recoating image, showing the capability of the proposed approach to detect the two metallization slivers.

Figure 14 shows the pre-recoating image and the post-recoating image, where the automated anomaly detection is highlighted in red, as done in the previous subsection. Figure 14 shows that the two local anomalies were correctly detected by the proposed approach, despite their quite small size. This result is an example of the potential suitability of the methodology in identifying not only macroscopic anomalies but also much smaller and local ones.

### 3.3. Influence of the Choice of the Transfer Function $f_{T}$

**Figure 15:**
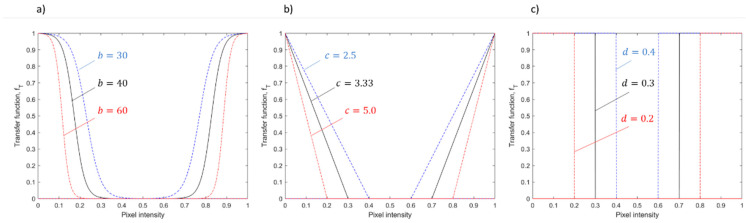
Three different functional formats for $f_{T}$ with varying parameters: (**a**) sigmoid function, (**b**) linear function, and (**c**) threshold function.

In order to determine the influence of the transfer function, $f_{T}$, and its parameters on the performance of the method, a sensitivity analysis was carried out with respect to parameter b in Equation (3). Parameter b is the one that determines the width of the pixel intensity range where the function $f_{T}$ is associated with an approximately null value. A larger value of b denotes a larger range of pixels having approximately zero intensity in $I_{tr*}$, as shown in Figure 15a. In addition to studying different choices for this parameter while maintaining the definition of the function $f_{T}$, two alternative approaches to define such a function were explored, namely, a linear function (Figure 15b) and a step function (Figure 15c).

By using a linear function, the pixel intensity drops from 1 to 0 linearly, instead of following a sigmoid pattern. This transfer function can be defined as follows:

(5) $$\begin{array}{l} f_{T}\left(i_{tr}\right)=1-ci_{tr}\;\mathrm{if}\;f_{T}\left(i_{tr}\right)\geq 0,\;0\;\mathrm{if}\;f_{T}\left(i_{tr}\right)<0,\;\;i_{tr}<0.5,\\ f_{T}\left(i_{tr}\right)=\left(c-1\right)+ci_{tr}\;\mathrm{if}\;f_{T}\left(i_{tr}\right)\geq 0,\;0\;\mathrm{if}\;f_{T}\left(i_{tr}\right)<0,\;\;i_{tr}\geq 0.5. \end{array}$$

In this case, increasing c has the same effect as increasing b in Equation (3).

Another alternative is the step function. This corresponds to an image binarization operation, as the output is an image where pixel intensities can be either 0 or 1. It can be viewed as a dual-threshold binarization operation, where pixel intensities are set to 0 if their original intensities are in the range $d\leq i_{tr}\leq 1-d$; otherwise, they are set to 1. In this case, parameter d can be varied. Increasing d has the same effect as decreasing c in Equation (5). In this case, no additional alarm rule is needed, as $I_{tr*}$ is a binary image, and pixels with intensity equal to 1 are automatically signaled as anomalies.

An example of anomaly detection in one layer resulting from the choice of different transfer functions, $f_{T}$, and from different choices of the associated parameters b, c, and d is shown in Figure 15. Additional results on the percentage of false alarms obtained for different functions and different choices of their parameters are shown in Table 3.

**Figure 16:**
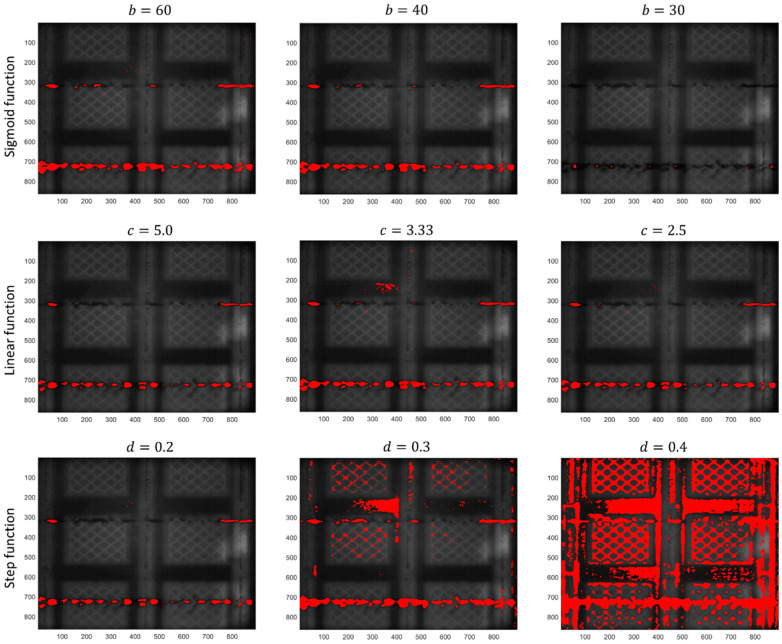
Example of outputs of the proposed approach with different definitions of the function $f_{T}$ and for different choices of its parameters.

Regarding the sigmoid function, a smaller value of b implies a less restrictive condition on the range of pixels whose intensity is set to approximately null values in $I_{tr*}$. The result is an image where the range of pixel intensities is wider even in the absence of actual powder bed anomalies, with a consequent increase in the limit $k_{\alpha }$ in Equation (4). By increasing such a limit, only pixels with the most extreme intensities that belong to actual anomalies can be detected. This results in reduced detection capability, as shown in the top right panel of Figure 16 (sigmoid function with b=30). By increasing the value of parameter b, the detection capability remains about the same for a large range of values of b. Indeed, as long as too small values are avoided, the performance of the method when a sigmoid function is adopted is quite robust and insensitive to the choice of parameter b.

A similar result was also obtained for the linear function. In this case, the range of values of c that lead to a good detection capability is even higher, although a slightly higher percentage of false alarms was observed, as shown in Table 3.

A quite different result was achieved by using the step function. As the output of the transfer function $f_{T}$ is a binary image, there is no need for the alarm rule defined in Equation (4). However, this prevents the user from having a control on the percentage of false alarms, leading to the results shown in the bottom panels of Figure 16. A reasonable result can only be achieved by setting the dual threshold very close to extreme intensities, i.e., close to 0 and 1, as in the case with d=0.2.

## 4. Discussion

The proposed method highlighted the suitability of in situ powder bed monitoring in EB-PBF using a high-spatial-resolution camera and no additional illumination source. Unlike other in situ sensing configurations proposed in the literature, this approach requires no extra time to perform the measurement, and it can rely on sensing equipment that is already available in industrial EB-PBF systems.

A preliminary investigation of the suitability of this methodology was discussed in [22], where a simple binarization of a transformed image obtained by subtracting the post- and pre-recoating images was proposed. In this study, we further extended and developed the automated anomaly detection methodology to make it more robust to false alarms, able to deal with both a lack of powder and an excess of powder anomalies, and more flexible with respect to part geometry variations that may affect the pixel intensity pattern in the monitored images.

The presented results highlight that the method is quite effective in detecting not only macroscopic discontinuities in the powder bed, but also small and local contaminations. As shown in Section 3, such discontinuities are particularly critical for the final quality of the part, as they directly affect the energy density provided by the electron beam to the material, with consequent generation of defects such as swelling and internal porosity. The performance of the method in terms of false alarms and justified alarms depends on the definition of the function $f_{T}$. The results presented in this study highlight that all tested functions can be effective choices provided that pixel intensities are filtered out within a sufficiently large interval of the normalized pixel intensity domain, e.g., between 0.2 and 0.8. If this interval is reduced, higher false alarm rates are expected. On the contrary, if this interval is enlarged, higher miss detection rates are expected. The linear and sigmoid functions resulted in higher robustness to false alarms; hence, they can be preferred in practice.

In this study, the anomalies inserted on purpose by damaging the powder recoating system produced a local excess of powder. In principle, the proposed approach is designed to also detect a lack of powder defects, but this kind of anomaly requires additional experimentation to be induced and tested. It is also worth pointing out that material remelting may heal the effects of powder bed anomalies. If an anomalous powder recoating occurs in one single layer (or a few consecutive layers), the effect on the quality of the part may be healed by consecutive remelting operations. If the anomaly persists for more consecutive layers, instead, the probability of inducing volumetric defects and/or geometrical distortions in the part is higher. From this perspective, the proposed approach may be combined with more conservative alarm rules that consider a minimum number of consecutive layers where a powder bed anomaly must be detected in a given location before an alarm is signaled.

Table 4 summarizes the potential link between powder bed anomalies and resulting defects in the part. A powder bed anomaly does not necessarily result in a defect, as the actual onset of defects depends on the severity of the anomaly, the persistence of the anomaly in consecutive layers, and the material properties and process parameters. However, Table 4 highlights the main quality issues possibly resulting from a nonhomogeneous powder bed, which motivated the study and development of powder bed monitoring methods in EBM.

As shown in Section 3, the proposed approach can be trained with a limited number of defect-free powder bed images, even acquired during the production of parts that are different from those in the build monitored herein. A training set is needed to estimate the empirical percentile of pixels intensities that can be used as a threshold to binarize the $I_{tr*}$ image. Since the $I_{tr*}$ image, by construction, includes only pixels whose intensity is 0 or close to 0, the effect of different geometries is mitigated, and the capability of distinguishing anomalies characterized by high values of $i_{th*}$ intensity is enhanced. Although a limited number of training images is sufficient (in this study, we used 20 images), an incremental extension of the training set, by including more and more powder bed images, assuming they were classified as anomaly-free, could further enhance the performance of the proposed approach.

In the presented approach, an off-axially mounted camera equipped with a sensor in the visible range and no optical filter was adopted. Nevertheless, possible extensions and improvements of the proposed approach can be explored by using a camera equipped with a near-infrared filter, to reduce the spectrum bandwidth and the dynamic range, and/or an external illumination source, to enhance the quality of the image and improve the characterization of the powder bed surface pattern.

## 5. Conclusions

Powder bed inhomogeneity is known to be a primary source of errors and defects in PBF processes, as it directly influences the volumetric energy density provided to the material with consequent local variations in the melting and solidification mechanisms. The automated detection of powder bed anomalies and discontinuities has been extensively investigated in L-PBF, and almost all industrial systems are equipped with powder bed cameras to this aim. Such capability is still lacking in EB-PBF, because of the different challenges imposed by the EB-PBF process on the use on in situ imaging. This study aimed to fill this gap with a first demonstration of the suitability of powder bed imaging combined with image processing and analysis techniques to automatically detect powder recoating errors in EB-PBF that have a detrimental effect on the final product quality.

The method is open to different possible extensions and improvements, in terms of both image processing algorithms and image acquisition equipment, including the use of ad hoc illumination sources or a selective use of the radiation spectrum to enhance the characterization of the powder bed surface. As a future development, powder bed monitoring in EBM can be further extended by exploiting different sensing methods to detect and characterize other phenomena of interest, such as surface powder oxidation [33,34], which is currently not feasible with the proposed approach.

## Figures and Tables

**Table 1:** Main camera specifications.

Sensor	Spatial Resolution	Integration Time	Field of View
CMOS sensor, grayscale, visible range	130 µm/pixel	0.04 s	About 90% of build area

**Table 2:** Estimation of false alarm metrics.

% of False Alarms	Average Extension of False Alarm Regions	Max Extension of False Alarm Regions
9.7 × 10^−5^	4 pixels	32 pixels

**Table 3:** Percentage of false alarms resulting from different definitions of the function $f_{T}$ and from different choices of its parameters.

	% of False Alarms		
Sigmoid function	b=60	b=40	b=30
	7.0 × 10^−5^	9.7 × 10^−5^	2.5 × 10^−6^
Linear function	c=5	c=3.33	c=2.5
	9.1 × 10^−5^	1.3 × 10^−4^	5.6 × 10^−4^
Step function	d=0.2	d=0.3	d=0.4
	5.2 × 10^−5^	4.4	58.7

**Table 4:** Potential effects of powder bed anomalies on the defectiveness of the manufactured part.

	Defects		
	Porosity	Geometrical Defects	Other
Lack of powder	Possible increase in over-melting porosity as a consequence of increased volumetric energy density	Swelling defects caused by intense remelting	Lack of powder lasting for multiple layers is expected to change the microstructural properties
Excess of powder	Lack of fusion pores as a consequence of decreased volumetric energy density	Swelling defects caused by a thicker solidified layer; possible delamination in case of very severe lack of fusion	Possible microstructural variations caused by decreased volumetric energy density
Contamination (large spatters, debris)	Possible lack of fusion pores as a consequence of beam attenuation/decreased volumetric energy density	Possible swelling defects in the presence of very severe contamination	

## Data Availability

The data presented in this study are available on request from the corresponding author.
